# Fine Mapping of Type 2 Diabetes Susceptibility Loci

**DOI:** 10.1007/s11892-014-0549-2

**Published:** 2014-09-20

**Authors:** Andrew P. Morris

**Affiliations:** 1 Department of Biostatistics, University of Liverpool, Duncan Building, Daulby Street, Liverpool, L69 3GA UK; 2 Department of Molecular and Clinical Pharmacology, University of Liverpool, Block A, Waterhouse Building, Brownlow Street, Liverpool, L69 3GL UK; 3 Wellcome Trust Centre for Human Genetics, University of Oxford, Roosevelt Drive, Oxford, OX3 7BN UK; 4 Estonian Genome Centre, University of Tartu, Riia 23b, 51010 Tartu, Estonia

**Keywords:** Fine mapping, Genome-wide association study, Re-sequencing study, Locus, Causal variant, Causal gene, Trans-ethnic meta-analysis, Credible set, Annotation

## Abstract

Genome-wide association studies of type 2 diabetes have been extremely successful in discovering loci that contribute genetic effects to susceptibility to the disease. However, at the vast majority of these loci, the variants and transcripts through which these effects on type 2 diabetes are mediated are unknown, limiting progress in defining the pathophysiological basis of the disease. In this review, we will describe available approaches for assaying genetic variation across loci and discuss statistical methods to determine the most likely causal variants in the region. We will consider the utility of trans-ethnic meta-analysis for fine mapping by leveraging the differences in the structure of linkage disequilibrium between diverse populations. Finally, we will discuss progress in fine-mapping type 2 diabetes susceptibility loci to date and consider the prospects for future efforts to localise causal variants for the disease.

## Introduction

Large-scale genome-wide association studies (GWAS) have proved to be an extremely successful approach to identifying loci contributing genetic effects to type 2 diabetes (T2D) susceptibility across ethnicities [1–5, 6•, 7•, 8•, 9, 10••]. These loci are typically represented by a common lead SNP, defined here to have minor allele frequency (MAF) of more than 5 %. This representation does not take account of the possibility of multiple association signals at the locus, each deriving from variants acting independently of each other or through haplotypes. The association signals usually extend over hundreds of kilobases because of linkage disequilibrium (LD) between common variants, and include multiple genes through which the effect on T2D susceptibility may be mediated. It has become common, therefore, to name these loci according to the gene mapping closest to the lead SNP, unless there is a more compelling candidate nearby. However, with the exception of loci such as *SLC30A8* and *KCNJ11-ABCC8*, where the causal variants and effector transcripts have been validated through functional studies [11, 12], these labels are effectively arbitrary and offer no insight into the mechanisms through which GWAS signals impact on T2D susceptibility. Consequently, there has been limited progress in defining the pathophysiological basis of the disease, and the promised translation of GWAS findings into clinical practice remains, as yet, unfulfilled.

To extend characterisation of T2D GWAS loci, the most comprehensive fine-mapping experiment requires an exhaustive catalogue of variation across the region and necessitates powerful methodology to integrate statistical evidence of association with regulatory and functional annotation to reflect our prior beliefs about the likely mechanisms through which variants will impact on disease risk. In this review, we will describe available approaches for assaying genetic variation across GWAS loci and discuss statistical methods to determine the most likely causal variants across the region. We will consider the utility of fine mapping in multiple ethnic groups by leveraging the differences in the structure of LD between diverse populations. Finally, we will discuss progress in fine-mapping T2D susceptibility loci to date and consider the prospects for future efforts to localise causal variants for the disease.

## Assaying Genetic Variation for the Fine Mapping of GWAS Loci

The success of GWAS for the discovery of loci contributing to complex human traits can be partly attributed to the realisation of the “common disease, common variant” hypothesis [13]. Under this model, the genetic component of T2D susceptibility is determined by many common causal variants throughout the genome, each with only modest effect on disease risk. International collaborative efforts, such as the HapMap Project Consortium [14, 15] and the 1000 Genomes Project Consortium [16, 17•], have demonstrated that common variants in a population are arranged on relatively few haplotypes within “blocks” of strong LD, which are located between recombination hotspots. Consequently, genotypes at the millions of common variants throughout the genome are strongly correlated with each other, and thus can be captured by a subset of “tag” SNPs. The most efficient GWAS genotyping products, therefore, have been designed to include these tag SNPs, maximising coverage of common variation, but reducing costs without a corresponding loss in power for detecting association with complex traits [18]. However, although this design is beneficial for the discovery of GWAS loci, it poses substantial challenges to the dissection of association signals for the purposes of fine mapping because (i) the causal variant(s) will not necessarily have been directly typed and (ii) multiple variants may demonstrate equivalent statistical evidence of association because of the LD between them.

### Targeted Re-sequencing

The most comprehensive approach to assaying genetic variation across T2D susceptibility loci is through targeted deep re-sequencing of the regions in large numbers of cases and controls [19]. In the absence of errors, this approach would be expected to provide a complete catalogue of common and low-frequency SNPs, rare variants and even private mutations, in a sample of sequenced individuals across a locus. There has much recent progress in the development of powerful statistical pipelines for the identification of variation and calling of genotypes from sequence data [20], maximising the likelihood of directly assaying the causal variant(s) at a locus and increasing the probability of observing lower-frequency haplotypes that might improve fine-mapping resolution by breaking down the LD between common SNPs. However, despite substantial improvements in the efficiency of “next generation” technologies, undertaking deep re-sequencing remains a considerable financial undertaking in the large sample sizes needed to localise the causal variants of modest effect size we expect for T2D susceptibility.

### Custom-Designed Genotyping

A less-expensive approach to assaying genetic variation across a locus begins by targeted re-sequencing of a subset of cases and controls. Genetic variation that is present in this “reference panel” could then be genotyped in the remaining individuals using a custom-designed array, incurring considerably lower costs than re-sequencing the entire sample. Further savings can be made by making use of reference panels obtained from publicly available large-scale whole-genome re-sequencing efforts, such as the 1000 Genomes Project [16, 17•], and can be used for variant identification. The first phase of this project incorporates genotypes at more than 30 million variants, genome wide, in 1,094 individuals from multiple ethnic groups, and is expected to provide a near-complete catalogue of genetic variation with MAF > 0.5 % across diverse populations [17•]. However, the disadvantage of this two-stage strategy is that genetic variation, which is not observed in the reference panel, will not be included on the array and will thus reduce the likelihood of assaying the causal variant(s), particularly if they are rare.

### The Cardio-MetaboChip

The Cardio-MetaboChip is a custom Illumina iSelect genotyping array of 196,725 variants designed to facilitate cost-effective discovery and fine mapping of cardiovascular and metabolic traits, including T2D [21]. Variants in 257 fine-mapping regions, including 34 established GWAS loci for T2D, were selected from reference panels from the HapMap Project [15] and a pilot release of the 1000 Genomes Project [16]. In these regions, the Cardio-Metabochip offers substantial improvements in coverage over traditional genotyping products, including more than 40 % of common SNPs (MAF ≥ 5 %) present in European haplotypes from the first phase of the 1000 Genomes Project [17•] and more than 25 % of low-frequency variants (1 % ≤ MAF < 5 %) [8•].

### Exome Re-sequencing and Array Genotyping

Some models of complex disease architecture hypothesise that a substantial proportion of causal variants for disease will alter protein function as opposed to regulation. Under this model, the most cost-effective strategy for fine mapping would focus on assaying coding variation, which can most comprehensively be achieved through whole-exome re-sequencing of cases and controls, and will provide a complete catalogue of protein altering variants across the genome. An alternative to this expensive re-sequencing strategy is to make use of exome array genotyping. The Illumina Human Exome BeadChip includes coding variants identified in a reference panel of more than 12,000 whole-exome and whole-genome sequences from multiple ancestry groups. The array also includes tags for previously reported lead SNPs in GWAS loci for a range of complex traits, including T2D. Consequently, the array can be used to investigate the hypothesis that common variant association signals in established T2D susceptibility loci are, in fact, explained by lower-frequency coding variants of larger effect in these regions, which would not have been well captured by GWAS genotyping arrays. The disadvantage of this approach is that the array is limited to genetic variation present in the reference panel, and thus is less likely to include rare coding variants and will not allow investigation of private protein altering mutations in cases and controls.

### Imputation

Imputation techniques [22, 23•] make use of a “scaffold” of SNPs from GWAS arrays to predict genotypes present on higher-density reference panels, derived from targeted re-sequencing in a subset of cases and controls or those made available from large-scale international efforts, such as the 1000 Genomes Project [17•]. The haplotype structure of the (phased) reference panel is used to infer the probability distribution of possible genotypes at each variant that is not present in the GWAS scaffold. Imputation can thus provide more complete coverage of genetic variation across a locus, without the need for re-sequencing or additional genotyping, but is limited to variants that are present in the reference panel. The most widely used imputation methods, IMPUTEv2 [24] and minimac [23•], minimise computational burden by “pre-phasing” the GWAS scaffold before inferring genotypes at variants present in the reference panel. Both methods make use of the same underlying model of LD between variants in the reference panel, and are effectively indistinguishable in terms of accuracy and run-time. For both methods, the quality of imputation depends on MAF, the density and allele frequency spectrum of the genotyping array from which the scaffold is derived, and the number of haplotypes and depth of sequencing in the reference panel, amongst other factors. The reference panel should also be well matched, in terms of ancestry, to cases and controls to maximise imputation quality, particularly for low-frequency and rare variants, which are more likely to be population specific [17•].

## Methodology for Fine-Mapping T2D Susceptibility Loci

The traditional approach to test for association of T2D susceptibility with a genetic variant, irrespective of whether it is assayed through re-sequencing, array genotyping or imputation, is to compare the frequencies of the three possible genotypes between cases and controls [25]. In this setting, the most flexible approach makes use of a logistic regression model, typically assuming an additive effect of each allele on the disease (i.e. a multiplicative effect on the odds ratio). The major advantage of this framework is that it can take account of potential non-genetic confounding risk factors as covariates, which for T2D susceptibility might include age, sex, and overall and/or central obesity, as measured by body mass index and waist-hip ratio, for example. In the same way, we can also include covariates that represent underlying population structure that might be confounded with disease status, which might include indicator variables of region of residence or eigenvectors from principal components analysis [26].

The strength of evidence in favour of association of T2D susceptibility with a genetic variant in this framework is most often measured by means of a *p* value, which corresponds to the probability of the observed (or more extreme) distribution of genotypes across cases and controls under the null hypothesis of no correlation with disease status. The smaller the *p* value, the less consistent are the observed data with the null hypothesis, and thus, the stronger the statistical evidence is in favour of association of the variant with disease. Consequently, *p* values have been used to rank variants across a locus in terms of their likely causal impact on T2D susceptibility. The disadvantage of this approach is that *p* values do not take account of the power of the association test [27] and thus are not sufficient to quantify how confident, statistically, we are that variants are causal for the disease. This problem is often overcome in GWAS analysis by excluding variants with low power, in particular those with low MAF and/or poor imputation quality. However, this is not advantageous for fine mapping, since our goal is to evaluate evidence of association for a comprehensive catalogue of variation across a locus.

An alternative measure of the strength of evidence in favour of association of a variant with T2D susceptibility, which takes account of the power of the test at the cost of additional modelling assumptions, is given by the Bayes’ factor [27, 28], the ratio of likelihoods of the observed distribution of genotypes in cases and controls under the alternative and null hypotheses. Exact evaluation of Bayes’ factors can be computationally demanding, but simple approximations can easily be calculated on the basis of association summary statistics and thus can be applied in the context of meta-analysis [29].

### Credible Sets of Causal Variants

Within the Bayesian modelling framework described above, we can assess the resolution of fine mapping at a locus by constructing a “credible set” of variants that are most likely to be causal on the basis of statistical evidence of association from Bayes’ factors [30••]. Credible set construction relies on two underlying assumptions: (i) the causal variant has been tested for association and (ii) there is a single causal variant at the locus. Variants are first ranked according to their Bayes’ factor in favour of association (from largest to smallest). Variants are then added to the 99 % credible set, by moving down the ranking, until the cumulative posterior probability of association exceeds 0.99. Credible sets can be interpreted in a similar way to confidence intervals in a frequentist paradigm and provide a quantitative assessment of the resolution of fine mapping across a locus in terms of the number of variants included and the genomic interval they cover. Variants in the credible set can then be prioritised for further investigation and functional experimentation.

One potential limitation of this approach to fine mapping is that the “information” that is available for each variant across a locus may not be equivalent. For example, on the basis of association summary statistics from a meta-analysis, data for some variants may not have been reported for all contributing studies, for example, if they fail genotyping or quality control. As a result, lower quality variants are less likely to be represented in the credible set than those that are reported in all contributing studies. Imputation can help in providing more uniform coverage by generating summary statistics for all variants in the reference panel, although quality may vary according to MAF and the genotyping array used to construct the GWAS scaffold. However, if we make the assumption that the effect of a variant on T2D susceptibility is homogeneous across studies, we can make use of the available information and re-weight Bayes’ factors to a uniform sample size across the locus.

### Assessing the Evidence for Multiple Association Signals at the Same Locus

The formal approach to assessing the evidence of multiple association signals at the same locus is through conditional analysis. Within the logistic regression framework described above, we can include the genotype at the lead SNP as a covariate in the model. Any residual association is derived from variants other than the lead SNP, and thus represents independent signals at the locus. This process is typically performed iteratively, including as an additional covariate in the model at each stage the variant with the strongest residual association, until the signal at the locus is fully explained.

A major limitation of the iterative scheme is that it cannot be easily applied in the context of meta-analysis. However, approximate conditioning, as implemented in the GCTA software [31•], can be applied to association summary statistics obtained from meta-analysis, without cohort-level information from the contributing studies. GCTA makes use of individual level genotype data from one contributing study as a reference to approximate the structure of LD across the locus, from which the covariance between allelic effects at multiple SNPs in the regression model can be estimated. Ideally, the reference study will be large and be representative of populations contributing to the meta-analysis. The robustness of the approximate conditional analysis should also be evaluated by considering multiple reference studies to assess the impact of differences in LD structure between populations on inference.

At present, there is no consensus on the appropriate threshold of significance for multiple, independent association signals (in terms of *p* values or Bayes’ factors). The traditional “genome-wide” significance threshold (*p* < 5 × 10^−8^) is too conservative because we are testing fewer variants and we already have prior evidence of association for the lead SNP at the locus from previous studies. The results of (approximate) conditional analyses can be used, in the same way as described above, to construct a credible set of variants for each association signal at a locus.

### Trans-Ethnic Fine Mapping

Assuming that a causal variant at a GWAS locus is shared across ancestry groups, substantial improvements in the resolution of fine-mapping can be achieved by combining GWAS from different ancestry groups through trans-ethnic meta-analysis, by leveraging differences in the structure of LD between diverse populations [32, 33]. As a consequence, we would expect that different sets of SNPs will be in strong LD with the causal variant in different ethnicities. Trans-ethnic meta-analysis, therefore, will reduce the credible set of variants at a locus to those that are in strong LD with the causal variant in *all* ancestry groups. African ancestry populations have less extensive LD because of less recent population bottlenecks and migration and thus are particularly powerful for fine mapping.

Differences in the extent of LD between ancestry groups will introduce heterogeneity in allelic effects on T2D susceptibility across ethnicities at non-causal variants, which cannot easily be accommodated in traditional “fixed-effects” meta-analysis. The MANTRA methodology [34••] for meta-analysis was developed to overcome this problem in the context of trans-ethnic fine mapping by allowing for heterogeneity in allelic effects between populations according to a model of “relatedness” between them. For example, two European ancestry populations will be closely related and thus would be expected to share similar LD structure across the locus and homogeneous allelic effects. On the other hand, much greater heterogeneity would be expected between European and African ancestry populations because of the significant differences in the distribution and extent of LD between them. Simulations have demonstrated that, by accounting for heterogeneity in allelic effects between ancestry groups in this way, MANTRA offers considerable improvements in fine-mapping resolution than fixed-effects meta-analysis [34••, 35].

MANTRA provides an assessment in favour of association of each variant with T2D susceptibility by means of a Bayes’ factor, and can naturally be used for the construction of credible sets, as described above. However, approximate conditional analyses cannot easily be applied in a trans-ethnic context, because LD structure varies across ancestry groups, and thus cannot be represented by a single contributing reference study.

### Incorporating Functional and Regulatory Annotation

Lead SNPs at T2D GWAS loci typically map to non-coding sequence and do not have a direct functional impact on disease susceptibility. One possible explanation for this observation is that lead SNPs are tags for coding variants that are not well represented on traditional GWAS genotyping arrays. It has become common, therefore, to interrogate high-density reference panels, such as those from the 1000 Genomes Project Consortium [17•], for coding variants that are in strong LD with the lead SNP, as an indicator of a potential functional mechanism through which T2D susceptibility is mediated. If studies have already been imputed up to dense reference panels, credible set variants can also be investigated for functional annotation. Support for these coding variants can be assessed through prediction of their likely functional consequences using algorithms such as POLYPHEN [36] and SIFT [37].

Insight into the likely transcript(s) at a locus through which an effect on T2D susceptibility is mediated through regulation of gene expression can be obtained from expression QTL (eQTL) studies, the results of which are often made available through a variety of public data resources [38, 39]. Consequently, it has become typical to search for evidence of association of lead SNPs with expression of flanking transcripts (usually *cis*-eQTLs, defined within 1 Mb up and downstream of the transcription start site). There is substantial heterogeneity in eQTLs for some transcripts across different cell types [40, 41], so the ideal experiment will focus on tissues most relevant to T2D susceptibility (for example, pancreatic islets and liver), although this may not always be possible. For each *cis*-eQTL, we can then assess the evidence for the coincidence of GWAS and expression signals by: (i) identifying the variant with the strongest association with expression (referred to as the eSNP) and (ii) performing conditional analysis to determine if the eSNP association can be explained by the lead GWAS SNP. Alternatively, if studies have already been imputed up to dense reference panels, credible sets can be interrogated for eSNPs in the same way.

The Encyclopedia of DNA Elements (ENCODE) Project Consortium [42–44] has facilitated comprehensive discovery and description of genes, transcripts, and transcriptional regulatory regions, as well as DNA-binding proteins that interact with regulatory regions in the genome. These include transcription factors, different versions of histones and other markers and DNA methylation patterns that define states of the genome in various cell types. By overlapping credible sets with these functional elements, we may gain insight into the mechanisms through which T2D-associated variants impact disease risk. Within the Bayesian credible set paradigm, these insights may offer an opportunity to develop prior models for likely causal variants on the basis of their functional or regulatory annotation from ENCODE, independent of the statistical evidence of association, which may further enhance fine-mapping efforts in newly discovered T2D susceptibility loci.

## Progress in Fine-Mapping T2D Susceptibility Loci

At the time of writing, more than 80 loci have been robustly associated with T2D susceptibility at genome-wide significance. These loci have primarily been discovered through GWAS efforts in populations of European descent [1–4, 8•], but lately in other ancestry groups, including East Asians [7•], South Asians [6•], Hispanics [5], African Americans [9] and, most recently, through trans-ethnic meta-analysis [10••]. For the vast majority of these loci, the causal variants and the transcripts through which their effects on T2D susceptibility are mediated, remain obscure. Consequently, there has been increasing interest in the fine mapping of T2D loci to fully appreciate the genetic architecture and mechanisms underlying disease susceptibility and to prioritise variants and transcripts for functional validation.

### Evidence for Multiple Association Signals at T2D Susceptibility Loci

There is increasing evidence that multiple association signals, derived from independent index variants or through haplotype effects, are relatively widespread at T2D susceptibility loci. Improved power to detect these effects has been achieved through larger sample sizes and meta-analysis, dense genotyping (for example in Metabochip fine-mapping regions) and imputation up to targeted genomic sequences and 1000 Genomes Project Consortium reference panels.

At the *CDKN2A-B* locus, the strongest association signal for T2D susceptibility maps to a narrow inter-genic recombination interval spanning less than 10 kb [1, 4, 8•]. Fine mapping of the locus was undertaken by imputation into 1,000 T2D cases and 1,048 controls from the Diabetes Genetics Initiative up to a reference panel consisting of: (i) pilot data from the 1000 Genomes Project Consortium and (ii) targeted re-sequencing of 47 individuals from the same population background [45]. This analysis revealed a haplotype mapping to the recombination interval that was defined by two partially correlated sets of variants, best represented by rs10757282 and rs10811661, which together better explain the association signal than any single SNP across the locus. Three of the four possible haplotypes were observed, including one high risk (frequency 30 %, odds ratio of 1.29) and one low risk (frequency 16 %, odds ratio of 0.72). This haplotype effect was replicated by the Wellcome Trust Case Control Consortium [30••] by high-density custom-designed array genotyping in 2,000 T2D cases and 4,000 population controls of European ancestry, including variants identified through targeted re-sequencing of 32 CEU individuals from the HapMap Project Consortium [14].

**Fig. 1:**
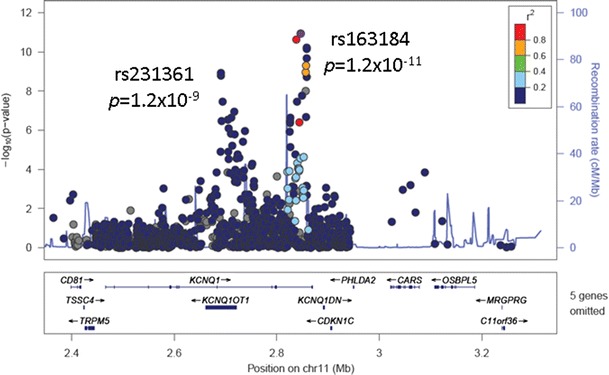
Fine-mapping of the *KCNQ1* locus. Each point represents a Metabochip SNP passing quality control in a meta-analysis of 34,840 T2D cases and 114,981 controls, primarily of European descent. Each SNP is plotted with their *p* value (on a −log_10_ scale, *y*-axis) as a function of genomic position (NCBI Build 36, *x*-axis). The lead SNP (rs163184) is represented by the *purple circle*. The *colour coding* of all other SNPs indicates LD with the lead SNP (estimated by CEU *r* ^2^ from the 1000 Genomes Project, June 2010 release): *red r* ^2^ ≥ 0.8, *gold* 0.6 ≤ *r* ^2^ < 0.8, *green* 0.4 ≤ *r* ^2^ < 0.6, *cyan* 0.2 ≤ *r* ^2^ < 0.4, *blue r* ^2^ < 0.2, and *grey r* ^2^ unknown. Recombination rates are estimated from the International HapMap Project and gene annotations are taken from the University of California Santa Cruz genome browser

At the *KCNQ1* locus (Fig. 1), meta-analysis of densely typed genetic variants from Metabochip fine-mapping regions in 34,840 T2D cases and 114,981 controls, primarily of European ancestry, revealed two signals of association at genome-wide significance that localised to different recombination intervals, represented by: rs163184 (*p* = 1.2 × 10^−11^), which maps to an intron of the gene, and rs231361 (*p* = 1.2 × 10^−9^), which resides in the *KCNQ1-OT1* transcript that controls regional imprinting [8•]. In the same study, nominal evidence (*p* < 10^−5^) of multiple association signals mapping to different recombination intervals was also observed at two additional loci: *DGKB* (rs17168486, *p* = 5.9 × 10^−11^; rs6960043, *p* = 3.4 × 10^−7^) and *MC4R* (rs12970134, *p* = 1.2 × 10^−8^; rs11873305, *p* = 3.8 × 10^−7^).

### Trans-Ethnic Fine Mapping

Large-scale trans-ethnic meta-analysis of GWAS was undertaken in 26,488 T2D cases and 83,964 controls from populations of European, East Asian, South Asian and Hispanic ancestry [10••], each imputed up to reference panels from the HapMap Project Consortium (Phase II/III) [14, 15]. The study validated previous observations that allelic effects on T2D at lead GWAS SNPs are predominantly homogeneous across ancestry groups [46], suggesting that susceptibility loci would be amenable to trans-ethnic fine mapping.

MANTRA was utilised to define 99 % credible sets of variants in ten established T2D susceptibility loci that demonstrated the strongest signals of association in the trans-ethnic meta-analysis. To assess the improved resolution offered by trans-ethnic meta-analysis, credible sets were constructed on the basis of: (i) European ancestry GWAS only and (ii) all GWAS, irrespective of ancestry. Improved fine-mapping resolution, both in terms of the number of variants included in the credible sets and the genomic intervals they covered, was observed at all but one of the loci after trans-ethnic meta-analysis. The greatest enhancement in fine-mapping resolution was observed at the *JAZF1* locus: the genomic interval covered by the credible set was reduced from nine SNPs (mapping to 76 kb) on the basis of European ancestry GWAS to just four SNPs (mapping to 16 kb) after trans-ethnic meta-analysis. The five excluded variants show strong LD with the lead SNP in European descent populations, but not in other ancestry groups (for example *r* ^2^ < 0.05 in CHB + JPT individuals from the HapMap Project Consortium [14]).

### Incorporating Functional and Regulatory Annotation

With the exception of loci such as *SLC30A8* and *KCNJ11-ABCC8*, the majority of lead SNPs at T2D susceptibility loci map to non-coding sequence. On the basis of reference panels from the 1000 Genomes Project Consortium [16, 17•], there has been little convincing evidence to date of coding variants that could explain the association signals of lead GWAS SNPs. One exception is at the *CILP2* locus, where *TM6SF2* E167K is in strong LD (*r* ^2^ = 0.95) with the lead SNP (rs10401969), although it is predicted to be tolerated by SIFT [37].

Investigation of potential effects of lead GWAS SNPs acting on T2D susceptibility through regulation of expression has proved to be more insightful. Examination of eQTL data for expression of transcripts in multiple tissues have provided compelling evidence of association of coincidence of lead GWAS SNPs and eSNPs for *IRS1*, *JAZF1*, *CAMK1D*, *KLF14*, *CCNE2*, *GRB14*, *ANK1* and *BCAR1*, providing evidence for mechanisms through which effects on T2D susceptibility are mediated at the corresponding loci [4, 8•].

The 99 % credible sets derived at GWAS loci on the basis of trans-ethnic meta-analysis [10••] were interrogated for overlap with sites of predicted regulatory function from the ENCODE Project Consortium [43]. Credible set variants were significantly enriched for overlap with sites of DNaseI hypersensitivity (*p* = 0.038) and transcription factor binding (*p* = 0.0060). At the *JAZF1* locus, one variant in the 99 % credible set (rs1635852) maps to a region of open chromatin with enhancer activity, which is bound by several transcription factors. This SNP has previously shown to have allelic differences in enhancer activity in pancreatic islets [47], and has been associated with *CREB5* expression, highlighting this as a potential effector transcript though which T2D susceptibility is mediated.

## Conclusion

The last 10 years have seen substantial advances in the discovery of loci contributing effects to T2D susceptibility, for which much of the success can be attributed to large-scale international collaborative efforts such as the DIAGRAM Consortium [4, 8•] and the AGEN-T2D Consortium [7•]. At the majority of these loci, the causal variants and transcripts have yet to be determined. However, initial fine-mapping efforts are providing the first invaluable insights into the genetic architecture and pathophysiological basis of the disease. Future fine-mapping efforts will be enhanced by improvements in the efficiency of re-sequencing technologies, facilitating large-scale targeted or whole-genome studies in large sample sizes. Higher-density reference panels including more individuals from more diverse population groups will improve the utility of imputation in fine-mapping studies, providing more complete coverage of genetic variation across ethnicities without the need for re-sequencing. Advances in statistical method development, incorporating improved understanding of the genome from the ENCODE Project Consortium [42, 43], will augment causal variant localisation and provide further acumen as to the mechanisms through which GWAS loci influence T2D susceptibility, with the ultimate goal of translation of these findings into clinical practice and the resulting public health benefits.
